# Notes on Preparations for the Sick

**Published:** 1905-09

**Authors:** 


### Notes on Preparations for the Sick.

---

**The Pasteur Filter** (Chamberland).—J. DEFRIES & SONS LTD., 146 Houndsditch, London, E.C.—One of the most serious problems that medical experts have been called upon to solve is the production of a filter for drinking-water capable of removing not only suspended inorganic matter, but also pathogenic organisms. Needless to say, many filters, though fulfilling the first requirement, are totally inadequate in the more important detail. The filter-tubes of the Pasteur-Chamberland type are composed of specially-prepared porcelain, through which it is claimed that bacteria cannot pass. Experiments have been made by numerous experts, and their results are highly satisfactory.

We believe that these filters are quite reliable, and that they do what is claimed for them. They should therefore be a true safeguard against waterborne disease.

---

**Mellin's Feeding Bottle.**—MELLIN'S FOOD LTD., Peckham, London.—The simplicity and efficiency of this arrangement of teat, patent valve, and bottle are too well known to need full description. It should be doing a great deal towards the reduction of infant mortality. The facility with which it can be cleansed, and the regulation of the dose for each meal at various ages, must greatly facilitate artificial feeding. We notice that the glass is of very indifferent quality, and that the bottles are made in France.

---

**Mist. Senecio Co.; Hewlett's Antiseptic Cream; Creosalgen Jelly; Laxans,** compressed tablets.—C. J. HEWLETT & SON, Charlotte Street, London, E.C.—The makers send us these special preparations, which all appear to be good and to serve the purposes intended.

The Senecio Compound is another of the numerous uterine tonics which are said to be useful in all varieties of uterine disorders.

The Antiseptic Cream is a compound of zinc oleate with boric acid, lanoline, &c., which should serve many useful purposes.

The Creosalgen Jelly is prepared with lanoline and 1 per cent. of surgical creosalgen. It is an ideal lubricant for use in midwifery practice, as it is a reliable antiseptic more powerful than carbolic acid, is devoid of caustic properties, and does not stain surgical apparatus.

The Laxans Tablets contain gr. iss. of phenolphthalein. They are a laxative of non-irritating character and medium strength.

---

**Hemaboloids; Formolyptol.**—THE PALISADE MANUFACTURING Co., New York. London: ANDRUS & ANDRUS, 46 Holborn Viaduct.—Hemaboloids are stated to contain nucleo-peptones, and to possess hematinic, lecithinic, and nucleogenic properties. Chemical analysis reveals the presence of organic iron, and also of non-coagulable proteid. The ash contains phosphoric acid, which is derived from organic phosphorus existing in the preparation. The flavouring is very pleasant, and is partly due to the presence of a little alcohol. The therapeutic agents present should be readily assimilable, and the iron should have no undesirable action. Hemaboloids are also prepared with the addition of arsenic and strychnine.

The Formolyptol is an antiseptic and germicidal fluid containing aceto-boro-glyceride (5 per cent.) with formaldehyde (0.2 per cent.) in combination with pinus pumilio, eucalyptus, myrrh, storax, and benzoin. It forms a slightly opalescent fluid, useful for throat spray, nasal douche, gargle, douches, and dressings of all kinds.

---

**Renine** (Eau nitrée d'Alsace.—Source César).—RENINE LTD., 254A High Holborn, London, W.C.—At the present time there are so many medicinal waters on the market, that it is somewhat difficult to advocate the claims of any special one without a prolonged trial. As far as chemical analysis can decide the value of a water, Renine should prove a desirable addition to already existing types. It is obtained from a spring probably used by the Romans, and contains moderate amounts of various mineral salts, including compounds of iron, calcium, magnesium, potassium, sodium, in combination with sulphuric, hydrochloric, and carbonic acids. The principal saline ingredient is nitrate of potassium, amounting to one-half of the total mineral matters. To this is probably due the fact that the water possesses well-marked diuretic properties.

---

**Chloroform.**—BURROUGHS, WELLCOME & Co., London.—It has recently been demonstrated that a proportion of ethyl chloride, so small as hardly to be capable of detection by chemical means, is often present in chloroform, and has a marked beneficial influence on its action as an anæsthetic. To overcome the difficulty occasioned by its variability, "Wellcome" Brand Chloroform has been introduced. This is particularly characterised by containing a small, but definite, amount of ethyl chloride. In its production the most scrupulous care is taken to ensure the highest attainable degree of purity, and especially freedom from irritating products of decomposition.

---

**Liq. Xenine Co.**—OPPENHEIMER, SON & Co., 179 Queen Victoria Street, London, E.C.—This is a new preparation, containing the active principles of rhamnus purshianus and leptandra virginica. It is free from all bitter resinous elements, but contains all the aperient principles of the two drugs; it should therefore stimulate the liver and intestinal tract, producing peristalsis without pain.

**Cocoid.**

| | |
|---|---|
| Hyd. c. Creta ... ... ... ... ... ... | gr. $\frac{1}{6}$ |
| Pulv. Rhei. Co. } Sodæ Bicarb. } ... ... ... | āā gr. $\frac{1}{2}$ |

This chocolate compound may be chewed without flavour or odour of the drugs becoming apparent. The old Gregory powder should be out of date in the presence of such elegant preparations, which no child or even the most fastidious patient could describe as in the least degree nauseous or unpleasant.

---

**Chologen Tablets.**—CHARLES YARROW & Co., Basinghall Street, London, E.C.—The remarkable reception accorded on the Continent to Dr. Robert Glaser's Chologen Tablets in gallstone and liver affections has induced the manufacturers to endeavour to extend their use in this country. "Chologen is a combination of very small doses of mercury with aromatics derived from the group of purgative and at the same time vegetable agents (podophylline) and carminative and anti-spasmodic aromatics and oils." The tablets are prepared in three different combinations: "Nos. 1 and 2 are specially laxative, anti-fermentative, anti-putrescent, and restrict inflammation. No. 3 has in addition a stimulating effect, owing to the presence of free camphor."

It is claimed that "with Chologen the need of a dreaded operation is only to be feared under exceptional conditions: thus a great triumph for a modern internal medicine is secured." There is evidence for and against any kind of medicinal treatment of gallstones. No remedy such as this can replace the interference of the surgeon in appropriate cases, although Dr. Glaser claims to have cured 76 per cent. of his cases by this means.

---

**Candol.**—THE CANDOL CO., 20 Eastcheap, London, E.C.—Candol is described as a desiccated malt extract possessing marked diastatic power. It is in a convenient form for administration, and it lacks the excessive sweetness of the ordinary extract. In the laboratory it was found to fulfil its requirements, being capable of converting starch into soluble maltose at the body temperature.

